# The complete mitochondrial genome of *Triplophysa nanpanjiangensis* Zhu and Cao 1988 (Cypriniformes: Nemacheilidae)

**DOI:** 10.1080/23802359.2023.2290119

**Published:** 2023-12-12

**Authors:** Jingzhuang Zhao, Li Zou, Lu Tian, Mingqiu Liu, Haibo Jiang, Zhonggui Xie, Zhiqiang Liang

**Affiliations:** a College of Animal Science, Guizhou University, Guiyang, Guizhou, China; b Hunan Fisheries Science Institute, Changsha, Hunan, China

**Keywords:** Mitochondrial genome, molecular phylogeny, Nemacheilidae, *Triplophysa nanpanjiangensis*

## Abstract

The genus *Triplophysa* is an ideal taxon for understanding geological evolution. In this study, for the first time, we report the complete mitochondrial genome of *T. nanpanjiangensis* Zhu and Cao 1988 using the Nanopore sequencing. It is a circular genome with a length of 16558 bp, comprising 22 tRNAs, 13 protein-coding genes (PCGs), two rRNAs, and one non-coding control region. The phylogenetic tree demonstrates that *T. nanpanjiangensis* is sister to *Triplophysa zhenfengensis* and placed within the genus *Triplophysa*. Our mitogenomic studies provide a new pathway for understanding the molecular phylogeny of the genus *Triplophysa*.

## 1. Introduction

The family Nemacheilidae is mainly distributed in Eurasian waters, and more than 600 fish species have been reported (Eschmeyer and Fong 2015). Its limited dispersal ability makes it an ideal taxon for understanding geological evolution (Chen et al. 2019). The genus *Triplophysa* is one of the most species-diverse taxa in the family Nemacheilidae, with 175 fish species having been reported (Eschmeyer et al. 2020). It is mainly distributed in lakes, rivers and streams on the Qinghai-Tibet Plateau and adjacent region (Wu et al. 2018). We sequenced the complete mitochondrial genome of *Triplophysa nanpanjiangensis* (Zhu and Cao 1988), which provides base data for the phylogeny of the genus *Triplophysa*.

## 2. Materials

**Figure 1.**
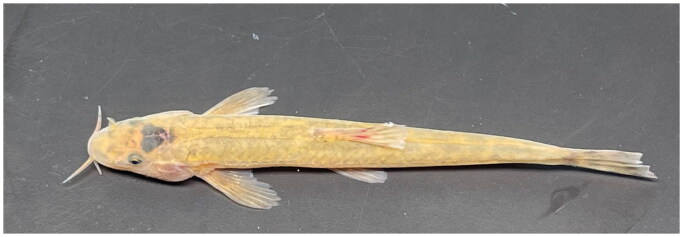
Species image of *T. nanpanjiangensis*. The most characteristic features of this species are dorsal fin III-7; anal fin III-5; pectoral fin I-9; ventral fin I-6; caudal fin 16; normal eyes; and caudal fin slightly forked. Photo taken by Zhiqiang Liang in changsha, China.

The fresh (living) *T. nanpanjiangensis* (Figure 1) were collected from Zanyi County, Yunnan Province, China (Longitude: 104.0039°E, Latitude: 25.6503°N), with were anesthetized using MS222, fixed in 75% alcohol. The voucher specimens (collector: Jingzhuang Zhao, 275848464@qq.com) were deposited in the Hunan Fisheries Science Institute (contact person: Zhiqiang Liang, liangzhiqiang@163.com) under the voucher number NO.HFSIF202302.

## 3. Methods

The total genomic DNA of *T. nanpanjiangensis* was extracted using the sodium dodecyl sulfate (SDS) method combined with a purification column. DNA concentrations were measured with a Qubit while DNA quality was assessed with a NanoDrop ND-1000 spectrophotometer (NanoDrop Technologies, DE, USA). The extracted DNA sample were DNA damage repaired and end-repaired. DNA was purified from the samples by AMPure XP magnetic beads (Beckman Coulter) before elution in DNA elution buffer. And the purified DNA was quantified using the Qubit fluorometer (Thermo). Then, the Ligation Sequencing Kit (SQK-LSK110) was used to perform adaptor ligation and library prepped. The library was loaded onto the R9.4 flow cell of the MinION sequencing device (Oxford Nanopore Technologies, Oxford, UK) and sequenced for 48-72 h. Following quality filtering, reads were assembled using SPAdes v3.11.1 with parameters that mainly included kmer values ranging from 27 to 127. MitoZ 2.3 was selected for genome annotation. Circos was used to generate the genome map (https://circos.ca/tutorials/lessons/).

We used the the complete mitogenome sequence of *Cobitis sinensis* (AY526868, Chen and Wang 2015) as an outgroup along with the following sequences of other species from the same Nemacheilidae family: *T. siluroides* (KJ781206, Chen et al. 2016), *T. siluroides* (KT213603, Wang et al. 2016), *T. pappenheimi* (KT213600, Wang et al. 2016), *T. robusta* (KM406486, Sun et al. 2016), *Barbatula minxianensis* (KT213596, Wang et al. 2016), *T. dalaica* (KT213590, Wang et al. 2016), *T. dorsalis* (KT241024, Lei et al. 2016), *T. scleroptera* (KT213602, unpublished), *T. chondrostoma* (KT213589, Wang et al. 2016), *T. markehenensis* (KT213594, Wang et al. 2016), *T. zhenfengensis* (MT992551, unpublished), *T. rosa* (JF268621, Wang et al. 2012), *T. xiangxiensis* (KT751089, Wang et al. 2017), *T. wangmoensis* (KX376479, unpublished), *Schistura incerta* (MK361215, Chen et al. 2019), *S. fasciolata* (MW192448, unpublished), *Yunnanilus niger* (OM681515, unpublished), *Oreonectes furcocaudalis* (KX778472, Luo et al. 2017), *Paranemachilus genilepis* (MT845213, Luo et al. 2020). Phylogenetic analyses were performed using the full mitochondrial sequence. Phylogenetic analyses applying maximum-likelihood algorithm were performed using IQ-TREE v2.1.3, with the GTR + F + I + G4 model and 1000 rapid bootstrap replicates.

## 4. Results

**Figure 2.**
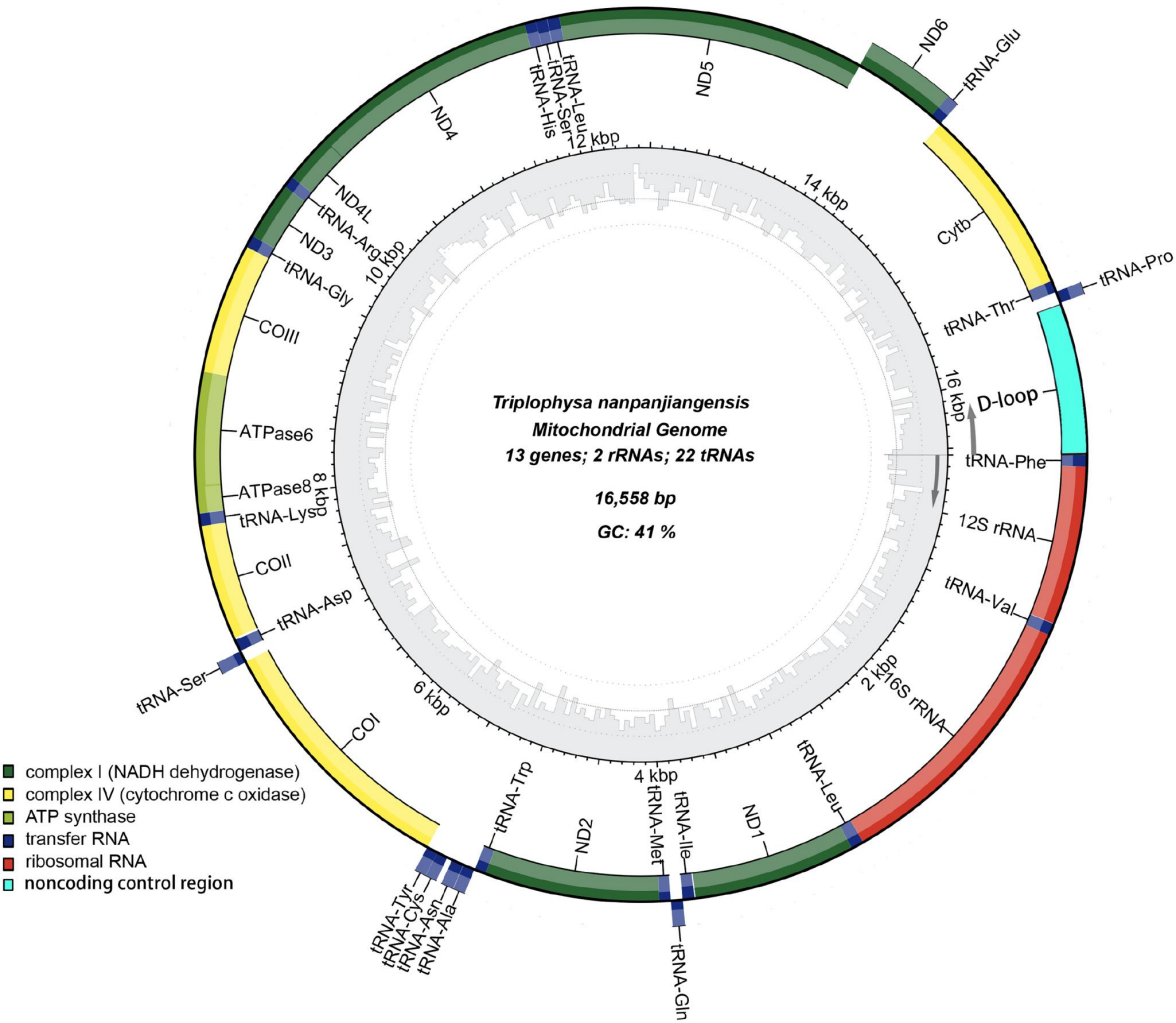
Map of the assembled T. nanpanjiangensis mitochondrial genome (GenBank Accession: OQ274895) consisting of 13 protein-coding genes (dark green, light green and yellow), 22 transferRNAs genes (dark blue), two ribosomal RNA genes (red), and one non-coding control region (D-loop, light blue). genes encoded on the reverse strand and forward strand are illustrated outside the circle and inside the circle, respectively. The inner ring displays the GC content of the genome.

The mitochondrial genome of *T. nanpanjiangensis* is 16558 base pairs (bp) in length with a content of 41% GC, the read coverage depth map is shown in Figure S1. It contains 13 protein-coding genes (PCGs) (*ND1-6*, *ND4L*, *COXI-III*, *ATP6*, *ATP8*, *Cytb*), 22 transfer RNA (tRNA) genes, two ribosomal RNA (rRNA) genes, and one control region (D-loop) (Figure 2). The rRNA and protein-coding genes (PCGs) were identified and confirmed *via* multiple sequence alignment with homologous genes from published mitochondrial genomes of other species in Nemacheilidae. This is the same distribution as the complete mitochondrial sequence of the *Triplophysa* genus (Wang et al. 2016; Lei et al. 2016). 12S-rRNA and 16S-rRNA were located between the tRNA^phe^ and tRNA^Leu^ genes and separated by the tRNA^Val^ gene. All tRNA genes can be folded into a typical cloverleaf structure with lengths ranging from 65 to 76 bp, which is comparable to *T. rosa* (Wang et al. 2012). The base compositions are 31.27% for A, 28.25% for T, 24.66% for C, and 15.82% for G. The large ribosomal RNA (lrRNA) is 1672 bp in length, and the small ribosomal RNA (srRNA) is 950 bp in length. For the 13 PCGs, one type of starting codon (ATG) and three kinds of stop codons (TAA, TA–, and T–) were observed (Table 1). The same patterns for codon use are common in published mitochondrial genomes of *Triplophysa* genus (Wang et al. 2012; Wang et al. 2017).

**Table 1.** Characteristics of the mitochondrial genome of *T. nanpanjiangensis.*

Element	From	To	Length (bp)	Start codon	Stop codon
tRNA^Phe^	1	69	69		
12S rRNA	70	1019	950		
tRNA^Val^	1022	1093	72		
16S rRNA	1094	2765	1672		
tRNA^Leu^	2766	2840	75		
*ND1*	2841	3815	975	ATG	TAA
tRNA^Ile^	3822	3892	71		
tRNA^Gln^	3891	3961	71		
tRNA^Met^	3963	4031	69		
*ND2*	4032	5076	1045	ATG	T–
tRNA^Trp^	5077	5145	69		
tRNA^Ala^	5148	5216	69		
tRNA^Asn^	5218	5290	73		
tRNA^Cys^	5322	5386	65		
tRNA^Tyr^	5387	5454	68		
*COI*	5456	7006	1551	ATG	TAA
tRNA^Ser^	7007	7077	71		
tRNA^Asp^	7079	7151	73		
*COII*	7165	7855	691	ATG	T–
tRNA^Lys^	7856	7931	76		
*ATPase8*	7933	8100	168	ATG	TAA
*ATPase6*	8091	8774	684	ATG	TAA
*COIII*	8774	9557	784	ATG	T–
tRNA^Gly^	9558	9630	73		
*ND3*	9631	9979	349	ATG	T–
tRNA^Arg^	9980	10049	70		
*ND4L*	10050	10346	297	ATG	TAA
*ND4*	10340	11721	1382	ATG	TA-
tRNA^His^	11722	11790	69		
tRNA^Ser^	11791	11857	67		
tRNA^Leu^	11859	11931	73		
*ND5*	11932	13770	1839	ATG	TAA
*ND6*	13767	14288	522	ATG	TAA
tRNA^Glu^	14289	14357	69		
*Cytb*	14362	15502	1141	ATG	T–
tRNA^Thr^	15503	15574	72		
tRNA^Pro^	15573	15642	70		
D-loop	15643	16558	916		

**Figure 3.**
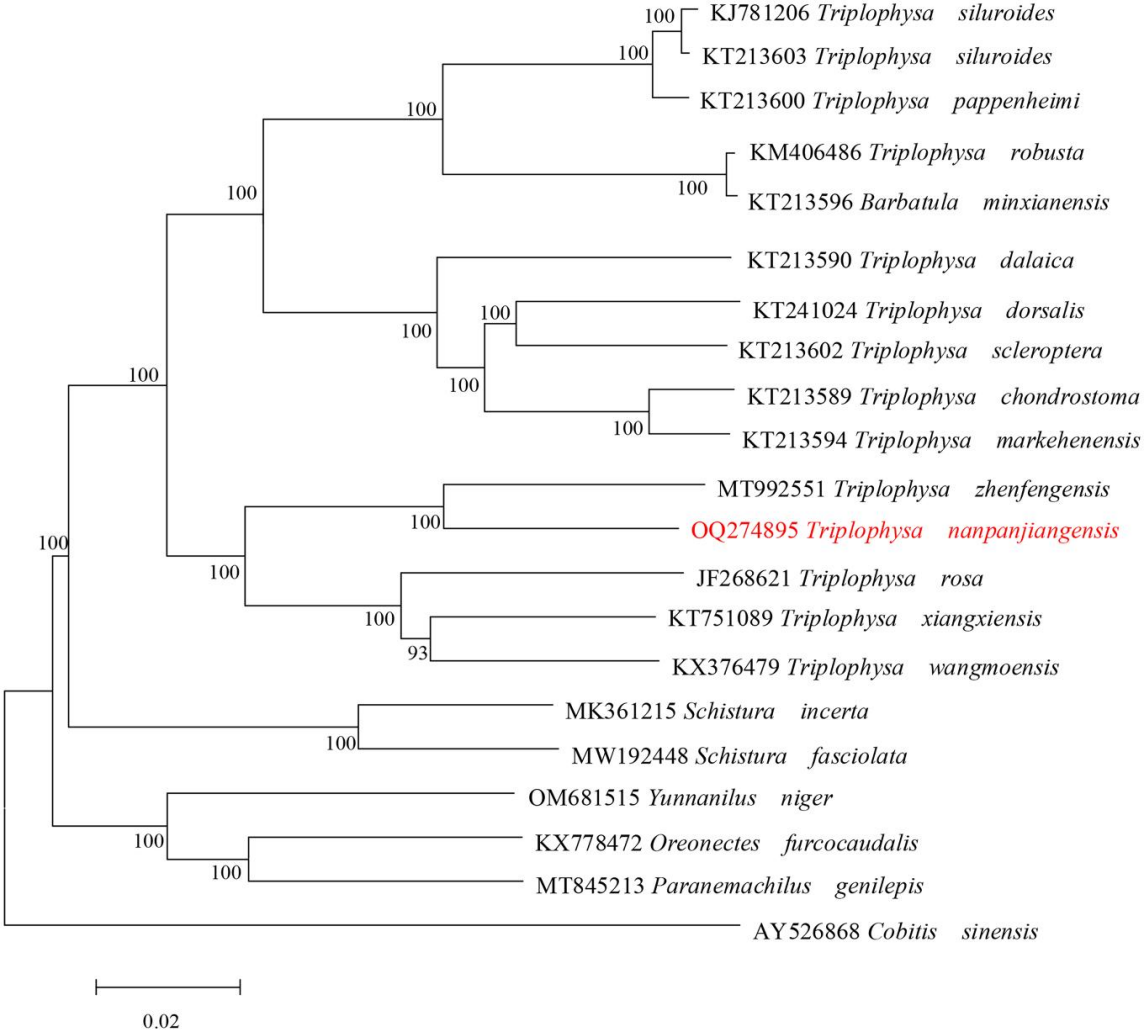
Maximum-likelihood (ML) phylogenetic tree was reconstructed based on the complete mitochondrial genome of *T. nanpanjiangensis* and other 20 Cypriniformes fishes. Accession numbers were indicated after the species names. Numbers at the nodes indicated bootstrap support values from 1000 replicates under the GTR + F + I + G4 model.

The phylogenetic tree showed that *T. nanpanjiangensis* was genetically closer to the genus *Triplophysa* than other species in Nemacheilidae (Figure 3). The results of the molecular phylogeny based on the mitochondrial genome were consistent with traditional morphological classification.

## 5. Discussion and conclusions

In the present study, we determined the complete mitochondrial genome of *T. nanpanjiangensis* for the first time. The phylogenetic tree supports *T. nanpanjiangensis* grouped with *T. zhenfengensis*, and clustered with other *Triplophysa* species. In short, the molecular data show that it should belong to *Triplophysa*, which proves that the current classification status is correct and valid.The gene species and distribution of this sequence are highly similar to those of other *Triplophysa* family species, Most of the genes were encoded on the H strand, except ND6 and eight tRNA genes encoded on the L strand (Chen et al. 2016; Sun et al. 2016). But, all PCGs in this sequence have one type of starting codon (ATG), which distinguishes them from other species in the *Triplophysa* family (Lei et al. 2016; Sun et al. 2017). It is expected that the mitogenome of *T. nanpanjiangensis* provided will contribute to future molecular species delimitation, assessment of the molecular evolution of the family Nemacheilidae and geoevolutionary studies.

## Supplementary Material

Supplemental Material

## Data Availability

The consensus genome sequence is openly available in GenBank of NCBI at [https://www.ncbi.nlm.nih.gov] under the accession no. OQ274895. The associated BioProject, SRA and BioSample numbers are PRJNA939422, SRR23646241 and SAMN33476296, respectively.
