# Supplementary figures and images for: The complete mitochondrial genome of Triplophysa nanpanjiangensis Zhu and Cao 1988 (Cypriniformes: Nemacheilidae)
Source: Mitochondrial DNA B Resour. 2023 Dec 12;8(12):1360–3. doi: 10.1080/23802359.2023.2290119 (PMC10776044; doi:10.1080/23802359.2023.2290119)

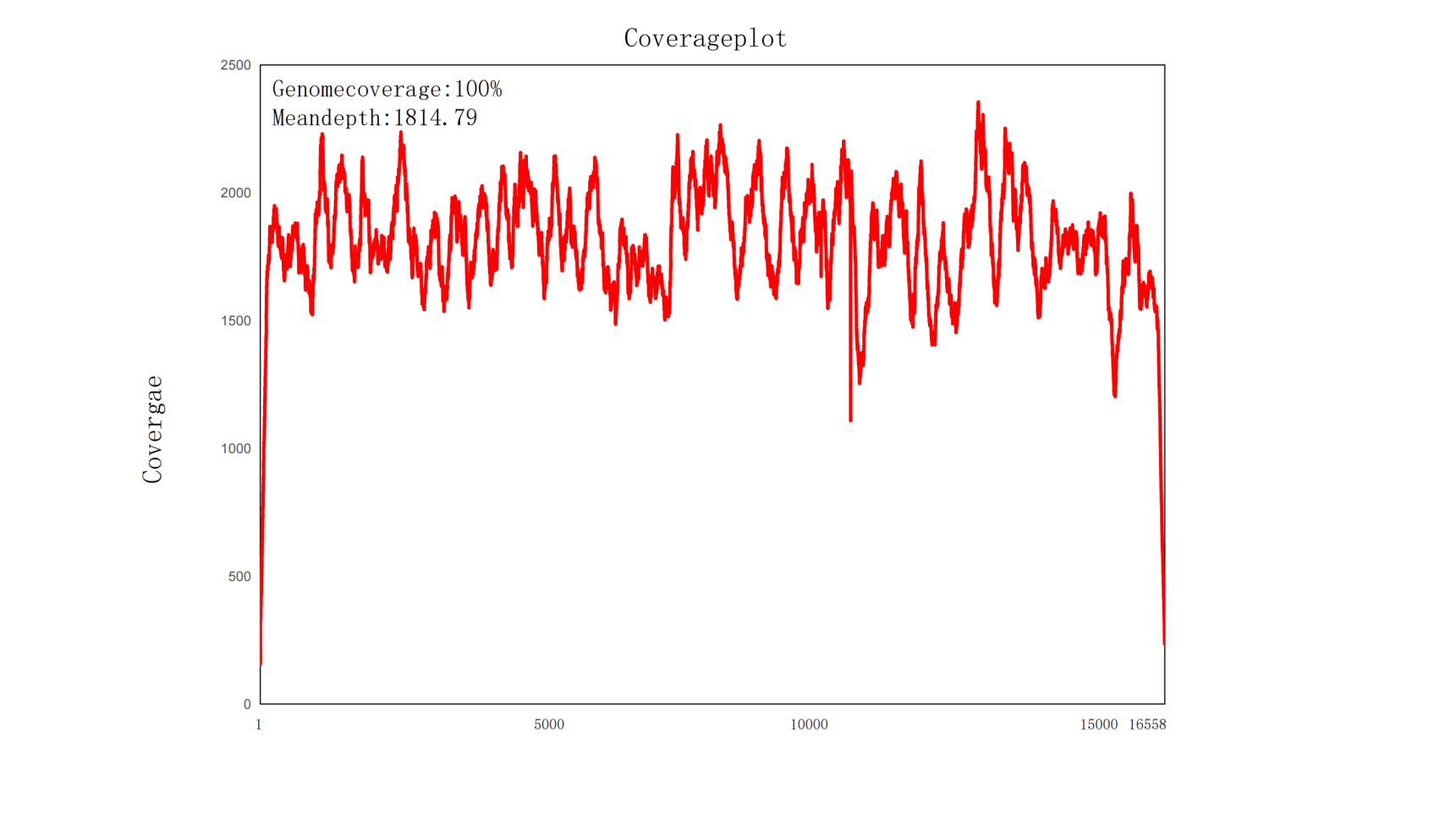


Figure S1. The read coverage depth plot.

Supplement: Supplemental Material [file TMDN_A_2290119_SM5309.docx]
